# Psychological Well-Being and Youth Autonomy: Comparative Analysis of Spain and Colombia

**DOI:** 10.3389/fpsyg.2020.564232

**Published:** 2020-09-25

**Authors:** Claudia Charry, Rosa Goig, Isabel Martínez

**Affiliations:** ^1^Department of Psychology, Santo Tomás University, Bogotá, Colombia; ^2^Department of Research Methods and Diagnosis in Education, National University of Distance Education (UNED), Madrid, Spain

**Keywords:** autonomy, psychological wellbeing, young people, Spain, Colombia

## Abstract

The construct of autonomy appears in literature associated with individual psychological wellbeing. In Ryff’s model, autonomy is presented as one of the dimensions of wellbeing, along with self-acceptance, positive relationships with others, environmental mastery, purpose in life, and personal growth. The present study compared the levels of autonomy and psychological wellbeing between Spanish and Colombian young people. Ryff’s Scale of Psychological Wellbeing and the Transition to Adulthood Autonomy (EDATVA) scales were used on a sample of 1,146 young people aged between 16 and 21; 506 Spaniards and 640 Colombians. Results showed differences in autonomy and in two of the four dimensions proposed by the EDATVA: self-organization and critical thinking. Similarly, important differences were observed in the subscales of positive relations and purpose in life. The importance of contextual factors in the development of psychological well-being and autonomy in young people in transition to adulthood is discussed.

## Introduction

Psychological well-being is a multidimensional and dynamic construct composed of a framework of dimensions where enjoying positive experiences and meeting basic needs are considered essential. Psychological well-being has been examined from multiple perspectives, and different academic fields have taken an interest in the construct due to its influence on other dimensions, such as individual performance, satisfaction levels, or the characteristics of interpersonal interactions (Gao and McLellan, 2018; Ryff, 2018, 2019). Psychological well-being involves subjective, social, and psychological dimensions, health-related behaviors, and practices that add meaning to an indiviual’s life and allow them to attain their maximum potential (Ryff, 2014; Ferrari et al., 2015; Lun and Bond, 2016; Friedman et al., 2017; Brim et al., 2019). Most researchers agree that well-being is a sign of an optimal psychological functioning that improves one’s life experience; therefore, it is understood as a set of factors that motivate people to pursue the satisfaction of their expectations (Crous, 2017; Maurya and Ojha, 2017; Bojanowska and Piotrowski, 2019). Yough (2017) stresses the individual’s personal circumstances in the context of well-being; people are unable to change these circumstances, for example, their country of residence or physical gender. Partaking from the relationship between psychological well-being and individual non-modifiable characteristics, different studies have analyzed the construct from different perspectives (Mota and Matos, 2015; Lun and Bond, 2016).

According to these studies, subjective psychological well-being is an important factor for human beings to achieve an optimal performance, that is, fully meeting one’s expectations in life; therefore, meeting expectations is frequently regarded as a predictive variable of positive individual development and is associated with high levels of overall well-being (McDowall, 2016; Reis et al., 2018).

The sociocultural context where a young person develops represents the universe of possible expectations that they can envision for their lives and the possible strategies that they can deploy to meet those expectations (Lacomba and Cloquell, 2017; Uribe et al., 2018). For instance, when comparing Spain and Colombia, the weight of the social support network appears to be higher in Colombia (Uribe et al., 2018). Therefore, psychological well-being is an idiosyncratic feature of each population, modulated by the visible types of occupations and interests that inspire the expectations of the individual (Güngör and Perdu, 2017; Alivernini et al., 2019; Mansoory et al., 2019; Klainin-Yobas et al., 2020). The context can also present special challenges, for instance the COVID-19 pandemic, characterized by the anxiety and fear of entire populations, especially for people with lower levels of autonomy and resilience (Koenig, 2020).

In Colombian youth, subjective psychological well-being has been associated with social interaction needs, often met within the immediate social context, whose additional function is to provide security during the transition to adulthood. These young people tend to develop hedonistic hobbies, such as watching television or listening to music, which are also used as distraction and evasion practices. During their transition to adult life, young people with higher levels of well-being begin to focus their energy on personal satisfaction and fulfillment, whereas young people with lower levels of psychological well-being tend to focus on social activities (Bahamón et al., 2019; Cabrera et al., 2019). Psychological well-being seems to be unrelated to gender, except for attitudes toward personal success, which suggests that coping strategies for men and women are similar but a certain sociocultural influence modulates gender-based roles and expectations (Blanco et al., 2019; Cabas et al., 2019).

In young Spaniards, psychological well-being is associated with the meaning of life and self-competence, both of which contribute to autonomy. Additionally, an adolescent’s adaptive capacity allows them to take a strategic approach toward their goals, which has been associated with high levels of intrinsic motivation (García, 2013; Mayordomo et al., 2016; Meléndez et al., 2018; García et al., 2019).

In general terms, two philosophical positions have guided psychological research on psychological well-being: hedonism, which focuses on happiness in life, and eudaimonism, centered on the enjoyment of significant experiences (Ryff, 2014; Yough, 2017). Different theoretical models of well-being have been proposed in accordance with these two philosophical positions. The hedonistic perspective emphasizes the evaluation of positive dimensions, such as satisfaction with life and positive affect (Ryff, 2019); the psychological well-being construct is built around the affective and cognitive evaluations of one’s life. On the other hand, in the eudaimonic perspective, the focus of attention is on intentional commitment, personal fulfillment, autonomy, and self-acceptance. These approaches diverge from each other, and consequently, the results of a given study will be presented from a specific angle (Ruini and Ryff, 2016; Ryff, 2018, 2019). Nevertheless, psychological well-being is usually quantified based on the interaction of the individual with positive and negative experiences (Weiss et al., 2016; Reis et al., 2018). Soenens et al. (2017) argued that the feelings of happiness and satisfaction with life are universal, although the sources of happiness and satisfaction can differ between societies and cultures.

Ryff (2018, 2019) proposed a theoretical model of psychological well-being comprising six different aspects of positive functioning: autonomy, environmental mastery, personal growth, purpose in life, positive relationships with others, and self-acceptance. Ryff’s six-factor psychological well-being model provides a comprehensive theoretical framework to analyze positive performance in young people (Sulimani-Aidan, 2016).

According to Sulimani-Aidan (2016) and Gao and McLellan (2018), research on psychological well-being has traditionally been conducted using a series of different variables, such as resilience, coping strategies, or capacity to adapt to difficult contexts, to establish possible associations. In this regard, Xi et al. (2018) have stressed the importance of having a purpose in life to achieve psychological well-being, which correlates with good physical and mental health during all stages of life. Hung and Appleton (2016) reported on the significance of formulating one’s purpose in life for young people in caregiving situations; the authors conclude that the possibility of achieving such an ideal becomes an engine of proactivity that motivates the individual toward development within his or her context using different skills connected with the achievement of the purpose, such as their ability to reflect on the problems that they face or to achieve autonomy.

In Ryff’s model, the definitions of autonomy and positive relationships with others correspond to the basic needs of autonomy and relationships for any individual (Gao and McLellan, 2018). According to Inguglia et al. (2015), autonomy is a fundamental dimension in shaping the psychological well-being of adolescents and young adults and is negatively correlated with loneliness and self-perceived isolation during this life stage. Parra et al. (2015) refer to autonomy as a key factor in a successful transition to adult life consisting of behaviors (individual capacity to act independently from others), cognitions (including self-efficacy, which empowers individuals to take action in different areas of their lives), and emotions (bonds built with others).

In the framework of family relationships during adolescence, there are at least three dimensions related to autonomy. The first dimension is behavioral, and it refers to the ability of a young person to act independently. The second dimension is cognitive, and it involves the acquisition of a sense of competence and agency that enables the person to decide how to take control of their lives. The third dimension is emotional; it refers to perceived independence in the form of self-confidence and individuality as well as the formation of new emotional links of increased symmetricity compared to those formed in childhood relationships (Parra et al., 2015; Soenens et al., 2017; Reis et al., 2018; Bojanowska and Piotrowski, 2019).

In the academic literature, autonomy is also positively associated with freedom, and negatively associated with the obstacles individuals face in order to fully enjoy their civil rights and participate in community life (Inguglia et al., 2015; Merrill et al., 2017; Vinayak and Judge, 2018; Carneiro et al., 2019). Hung and Appleton (2016) and Van der Kaap-Deeder et al. (2015, 2017) highlight the roles played by environmental conditions and social agents to encourage self-determination as a prerequisite to achieve autonomy. In an adverse context, where individuals experience deficiencies or inequalities with respect to others, community tools can eradicate these shortfalls by generating spaces for dialog and information in which young people can identify advantages or, at least, strengths to exploit in order to maximize their capacity to act toward the achievement of their own goals.

The first endorsement of autonomy resides in the social rights that come with being part of a community; they are defined and protected by the legal system and, therefore, associated with the enactment of citizenship (Balluerka et al., 2016; Krys et al., 2019). Therefore, autonomy acquires a political and social dimension associated with the mechanisms that guarantee the possibility of exercising self-determination in society; being aware of such rights is a first step (Hung and Appleton, 2016).

In this regard, the multiple relationships built by individuals and the infinite possibilities for experimentation allow for the development of commitment with one’s expectations and the increase of psychological well-being, two dimensions that have an effect on an individual’s ability to overcome personal challenges (Gaxiola and Palomar, 2016; Maurya and Ojha, 2017; Vinayak and Judge, 2018; Dutra-Thomé et al., 2019).

Sulimani-Aidan (2016) considers that social adjustment is conditioned by one’s future expectations in life and perceived self-efficacy, which facilitate the assumption that one’s behaviors will have an effect on subsequent success; therefore, young people who have positive beliefs about their academic and employment outlook adopt behaviors that favor self-fulfillment. These traits become protective and motivating factors that support people’s drive toward achievement and increase their psychological well-being in the future (Glynn et al., 2016; Crous, 2017; Dickens, 2018). Kaya et al. (2019) highlighted the influence of gender roles and the individual’s willingness to assume them as conditioning factors to their ability to adjust to their environment. For these authors, the current discussion on gender roles among men is centered around their refusal to interpret these roles in the normative sense relayed by their culture, which translates into a conflict that affects their level of psychological well-being. In this context, studies that fail to include the analysis of this impact may be missing on its explanatory power to understand psychological well-being.

According to Ryff (2018) and Kaya et al. (2019) the nature of a person’s transition into adulthood affects their psychological well-being, but also events that have an adverse effect on this process, supporting factors, and personality variables; for instance, young people who are open to experiencing adulthood as a period of expansion, maintain positive relationships with their environment, are outgoing, and have set personal goals tent to be successful in facing this stage. Additionally, self-esteem has been associated with a higher level of autonomy and with higher psychological well-being. Similarly, Skowron et al. (2009) suggest that psychological well-being determines young people’s success during the transition; Mota and Matos (2015) consider it essential for young people to develop resilience as a mechanism to improve their preparedness to face the challenges of adult life, when their ability to adapt to new environments and face vital challenges is crucial. Merrill et al. (2017) have identified the comprehension of other people’s challenging experiences as models that help one’s interpretation of our own transition to adult life. These experiences, often consisting of stories relayed by parents to their children, can be unknown for people who are raised in unstructured environments, which could restrict their ability to achieve psychological well-being (Kouros et al., 2017; Gao and McLellan, 2018).

In this context, the present study sought to compare psychological well-being and autonomy between groups of Spanish and Colombian young people as fundamental aspects for an adequate transition to adulthood.

## Materials and Methods

The present study used a descriptive-correlational approach. It was approved by the ethics committee of the participating universities. The results of the study are part of a larger research project carried out by the National University of Distance Education (Madrid, Spain) and Saint Thomas Aquinas University (Bogotá, Colombia).

### Specific Goals

The purpose of the present study was to examine possible differences between Colombian and Spanish youths in terms of psychological well-being based on the dimensions proposed by Ryff’s model and the construct of autonomy, but in the latter case not only based on Ryff’s definition, but also involving reflection and decision-making processes focused on oneself and on other people. Differences between boys and girls in Spain and Colombia are presented and analyzed and the scores obtained by participants from both countries are compared by schooling, employment, and state protection status.

### Participants

A total of 506 Spanish and 640 Colombian youths, selected by convenience sampling, participated in the study. A total of 34 Spanish and Colombian institutions were contacted, including educational institutions of different types, protection institutions, and companies, in order to observe the different conditions young people in this age range could face. Inclusion criteria included being within the age range established for the study, that is, between 16 and 21 years of age, as well as having basic reading and writing skills to respond to the instruments. The mean age for the sample of Spanish young people was 17.66 (SD = 1.6), and 18.69 for the Colombian group (SD = 1.8). Among the participants from Spain, 343 (67.8%) were girls and 163 (32.2%) were boys, and participants from Colombia were 347 (54.2%) girls and 293 (45.8%) boys.

### Instruments

Participants responded to a scale designed to measure autonomy during the transition to adult life (EDATVA, Bernal et al., 2019a) intended for young people between 16 and 21 years of age. The scale indicates an estimated degree of autonomy during the transition to adult life. It consists of 19 items grouped in four dimensions: self-organization (involving cognitive, organizational, and planning exercises focused on the subject); comprehension of context (including cognitive, organizational, and planning exercises, but now with respect to broader systems); critical capacity (the subject’s ability to define their position and defend their own interests), and socio-political involvement (understanding the consequences of one’s decisions on other systems and making decisions that take into account social responsibility). The EDATVA uses a response four-category response scale from 1 (*I completely disagree*) to 4 (*I completely agree*). Intermediate values (2 and 3) have no assigned labels. The score for each dimension is obtained by adding the direct scores obtained for the corresponding items (there are no inverse items), and performing a conversion as described by the authors in the scale’s manual (Bernal et al., 2019b). The final score is obtained by adding these transformed values. The instrument showed excellent psychometric qualities, including a Cronbach’s alpha of 0.84 for the total scale, 0.80 for the self-organization dimension, 0.74 for context comprehension, 0.70 for critical capacity, and 0.77 for sociopolitical involvement (Bernal et al., 2019a).

Additionally, participants responded to Ryff’s Psychological Well-Being Scale (Díaz et al., 2006). This instrument sought to evaluate psychological well-being based on the multidimensional model proposed by Ryff, and its psychometric properties were evaluated in adults, elderly adults, and adolescents (Ryff, 1989a,b, 1995; Van Dierendonck, 2005; Vleioras and Bosma, 2005; Fernandes et al., 2010). This scale consists of 39 questions grouped in six dimensions: self-acceptance (recognition and acceptance of one’s positive and negative traits), positive relationships (presence of close and stable relationships), autonomy (self-regulation of opinions and decision-making), control of the environment (management of day-to-day responsibilities), personal growth (creating conditions to develop one’s potential and evolve), and purpose in life (ability to clearly define life goals). Although the Well-Being Scale has a set of items aimed at assessing autonomy, its approach is mainly intra-subjective, focused on the person’s internal processes, whereas the EDATVA takes into account the inter-subjective psychological dimension, that is, the systems in which people interact. Items are answered on a six-category scale in the well-being scale: 1 (I totally disagree), 2 (I disagree), 3 (I partially disagree), 4 (I partially agree), 5 (I disagree), and 6 (I totally disagree). The final score is obtained by adding the values obtained for the 39 items, considering 17 inverse items distributed among the instrument’s six dimensions. The values of corresponding items are added to obtain the score of each dimension, and inverse items are recoded. The scale presents internal consistency levels between 0.68 and 0.83 (self-acceptance 0.83, positive relationships 0.81, autonomy 0.73, environmental mastery 0.71, personal growth 0.68, and life purpose 0.83) (Díaz et al., 2006).

### Procedure and Data Analysis

Different public and private institutions were contacted to obtain official authorization and validate the participants’ informed consent forms, as well as their parents’ in the case of underage participants. Electronic and hard copy versions of the instruments were created, which were administered considering the pertinence of each case and the participants’ access to electronic media and the internet. Both scales were administered in a single session. In some cases, the instruments were administered to groups and in other cases individually, depending on the participants’ schedules and availability of physical spaces.

Data were analyzed using descriptive statistics, and multivariate analysis of variance (MANOVA) was carried out to obtain the desired comparisons between the groups of young people from both countries, focusing on the constructs of well-being and autonomy; for this purpose, we evaluated normality and homogeneity of data variance. Univariate normality was evaluated using the Kolmogorov–Smirnov test; *p* < 0.05 values were obtained, which indicated the absence of normality for most variables. However, given the robust nature of the technique with respect to type I error and effect size, the size of the sample, and the similar size of the Spanish and Colombian groups (when the *n* value of the larger group was divided by the *n* value of the smaller group, the result was smaller than 1.5), MANOVA was maintained (Pituch and Stevens, 2016). Considering the lack of data normality, Levene’s median-based test was employed to assess variance homogeneity. Variance inhomogeneity was observed in most well-being subscales of the well-being test and in two of the EDATVA subscales (Levene test: *p* < 0.05). Also in this case, MANOVA showed robustness to the violation of the assumption as long as the size of the groups were the same. For this reason, as suggested by different authors (Nimon, 2012; Pituch and Stevens, 2016), random sampling (using SPSS software) was used in the largest group (*n* = 506, Colombia) in order to compare the groups. MANOVA was carried out separately for the dimensions of each scale, along with their respective total scores, based on the construct of similarity. In order to evaluate differences between the groups at the subscale level and in the total score of each instrument, multiple comparisons were made using a *t*-test for independent samples and Mann–Whitney *U* test, depending on the case; the Bonferroni correction was used to control for type I error (Huang, 2020).

## Results

Table 1 shows the means obtained for each group in each dimension of the well-being scale. The main similarities between the groups are related to the dimensions of personal growth and self-acceptance. The total mean of the well-being scale for the sample of Spanish young people was 171.83 (SD = 26.4), and 168.73 for the Colombian group (SD = 24.4). The MANOVA test was used to analyze the scores obtained by the two groups in the different subscales of the well-being scale (dependent variables), whose independent variable was the country to which they belonged. The multivariate contrasts obtained by Wilks’ lambda showed that the country has a statistically significant multivariate effect on the linear combination of the subscales composing the instrument (Λ = 0.83; *F* = 33.860; *p* = 0.00, partial square eta = 0.17). These results suggest the possible relationship between the country and the psychological well-being construct, in accordance with the model proposed by Ryff. The unstandardized discriminant function coefficients for the first multivariate combination are reported in Tables 1, 2 presents correlation indices between dependent variables.

**TABLE 1:** Comparisons between Colombian and Spanish young people by Well-Being Scale and EDATVA dimension.

**Instrument**	**Dimensions**	**Spain**	**Colombia**	***p***	Effect size*	***W*_*S*_**
Well-being scale	Self-acceptance	25.22 ± 6.13	25.19 ± 5.36	0.303	–0.033	0.004
	Positive relationships	27.62 ± 6.09	23.88 ± 5.58	0.000**	–0.334	0.176
	Autonomy	34.64 ± 7.24	33.89 ± 6.32	0.043	–0.064	0.008
	Control over the environment	24.81 ± 5.25	25.85 ± 4.82	0.011	0.080	–0.103
	Personal growth	32.96 ± 5.10	32.50 ± 5.33	0.174	–0.043	0.039
	Purpose in life	26.37 ± 5.78	27.71 ± 5.49	0.001**	0.107	–0.070
	Total score	171.83 ± 26.4	168.73 ± 24.4	0.020	–0.073	
EDATVA	Self-organization	19.07 ± 3.63	20.41 ± 3.20	0.000**	0.172	0.190
	Context analysis	20.64 ± 3.52	20.04 ± 3.48	0.001	–0.109	–0.209
	Critical thinking	18.40 ± 4.17	20.01 ± 3.63	0.000**	0.191	0.190
	Sociopolitical involvement	13.49 ± 4.68	13.65 ± 4.59	0.829	0.007	–0.048
	Total score	71.80 ± 10.95	74.02 ± 10.47	0.002**	0.097	

*Rosenthal’s R. **Significant per Bonferroni’s adjustment. *W*_*S*_ Coefficients from first unstandardized discriminant function.

**TABLE 2:** Bivariate correlation coefficients between the well-being scale subscales.

	**Self-acceptance**	**Positive relationships**	**Autonomy**	**Control over the environment**	**Personal growth**	**Purpose in life**
Self-acceptance	1	0.464**	0.440**	0.655**	0.451**	0.670**
Positive relationships		1	0.324**	0.384**	0.306**	0.310**
Autonomy			1	0.424**	0.399**	0.357**
Control over the environment				1	0.471**	0.691**
Personal growth					1	0.521**
Purpose in life						1

**p < 0.01.

Table 1 shows the average scores obtained by Colombians and Spanish young people in each EDATVA dimension. The mean overall EDATVA score for the Spanish group was 71.80 (SD = 10.95), and 74.02 (SD = 10.47) for the Colombian group. In general, MANOVA results for the EDATVA subscales showed an important effect of the country variable on overall autonomy (Λ = 0.89; *F* = 28.309, *p* = 0.00, partial square eta = 0.10). The unstandardized discriminant function coefficients for the first multivariate combination are reported in Tables 1, 3 shows the correlation indices between EDATVA subscales.

**TABLE 3:** Bivariate correlation coefficients between the EDATVA scale subscales.

	**Self-organization**	**Context analysis**	**Critical thinking**	**Sociopolitical involvement**
Self-organization	1	0.319**	0.305**	0.285**
Context analysis		1	0.349**	0.254**
Critical thinking			1	0.289**
Sociopolitical involvement				1

**p < 0.01.

Multiple comparisons using the Mann–Whitney *U* test were made between the Colombian and Spanish groups for the different subscales of the instruments. Table 1 shows the results of these comparisons and the effect sizes for each case. Statistically significant differences are observed in the positive relationships subscale, where Spanish participants obtained a higher mean than Colombian participants (see Table 1), as well as in purpose in life, in which Colombian participants scored higher than Spanish participants. Concerning the EDATVA, significant differences were found in the dimensions of self-organization, critical capacity, and overall scale score; in the three cases, Colombians obtained higher scores than Spaniards (see Table 1).

Comparisons by sex between Spaniards and Colombians were made using the Mann–Whitney *U* test as a function of sex (again, considering the lack of data normality), and important differences were observed among boys in the subscale of positive relationships in the well-being scale, where the mean for Spaniards was higher than for Colombians, as well as in EDATVA’s self-organization and critical capacity dimensions, in which Colombians presented higher average values than Spaniards (see Table 4). A significant difference in the positive relationships subscale was also found among girls (highest mean in Spanish groups), as well as in the purpose-in-life subscale, where the Colombian youth group showed a higher mean. Significant differences between Spanish and Colombian girls were found in almost every EDATVA dimension; Colombians showed higher mean scores in self-organization and critical thinking, and Spaniards presented the highest context analysis mean.

**TABLE 4:** Comparison by sex between Spaniards and Colombians for the different subscales of the instruments used.

**Sex**	**Instrument**	**Dimensions**	**Spain**	**Colombia**	p***	Effect size*
Boys	Well-being scale	Self-acceptance	25.76 ± 6.16	25.22 ± 5.38	0.200	–0.060
		Positive relationships	27.29 ± 5.99	23.80 ± 5.42	0.000**	–0.279
		Autonomy	34.29 ± 7.31	33.88 ± 6.52	0.490	–0.032
		Control over the environment	24.85 ± 5.37	25.83 ± 4.98	0.151	0.067
		Personal growth	31.74 ± 4.87	32.01 ± 5.35	0.472	0.034
		Purpose in life	26.23 ± 5.78	27.47 ± 5.40	0.034	0.099
		Total score	170.17 ± 26.93	168.22 ± 24.75	0.306	–0.048
	EDATVA	Self-organization	18.69 ± 4.13	20.25 ± 3.20	0.000**	0.166
		Context analysis	19.85 ± 4.26	20.02 ± 3.55	0.721	–0.017
		Critical thinking	17.86 ± 4.72	19.82 ± 3.76	0.000**	0.200
		Sociopolitical involvement	13.59 ± 4.79	13.52 ± 4.71	0.709	–0.017
		Total score	70.00 ± 12.81	73.63 ± 10.68	0.005	0.130
Girls	Well-being scale	Self-acceptance	24.99 ± 6.14	25.16 ± 5.25	0.730	–0.013
		Positive relationships	27.79 ± 6.13	23.95 ± 5.75	0.000**	–0.326
		Autonomy	34.87 ± 7.17	33.90 ± 6.24	0.018	–0.090
		Control over the environment	24.82 ± 5.23	25.87 ± 4.74	0.021	0.088
		Personal growth	33.58 ± 5.12	32.92 ± 5.31	0.067	–0.070
		Purpose in life	26.46 ± 5.90	27.91 ± 5.38	0.002**	–0.072
		Total score	172.54 ± 26.26	169.73 ± 24.13	0.058	–0.013
	EDATVA	Self-organization	19.25 ± 3.51	20.55 ± 3.14	0.000**	0.184
		Context analysis	21.02 ± 3.23	20.06 ± 3.49	0.000**	–0.145
		Critical thinking	18.65 ± 3.98	20.17 ± 3.63	0.000**	0.187
		Sociopolitical involvement	13.42 ± 4.64	13.76 ± 4.65	0.368	0.034
		Total score	72.35 ± 10.47	74.54 ± 10.78	0.013	–0.189

*Rosenthal’s R for all cases except total EDATVA score for women. Cohen’s *d* was used. **Significant per Bonferroni’s adjustment. ***Mann–Whitney *U* test for all cases except total EDATVA score for women. *t*-test applied for independent groups.

Different contrasts were found when considering differences in autonomy and well-being among young Spaniards and Colombians depending on whether they were studying, working, or receiving state welfare. A total of 317 Spanish participants (62.4%) were studying, 107 (21.1%) were working, and 84 (16.5%) were living on state welfare; in the Colombian sample, 443 (69.2%) participants were studying, 140 (21.9%) were working, and 57 (8.9%) were living on state welfare.

Table 5 shows that Colombians obtained significantly different means when compared with Spaniards in terms of purpose in life, self-organization, and critical thinking, while Spaniards obtained the highest and most significant mean score in the positive relationships subscale. Among participants who were working, significantly higher scores for the Colombian sample were observed in the critical thinking and self-organization subscales and in total EDATVA scores; for the Spanish sample, the positive relationships subscale (well-being scale) Spaniards showed the highest mean, a statistically significant difference as compared to Colombians. None of the instruments detected statistically significant differences when comparing Spaniards and Colombians who lived on welfare.

**TABLE 5:** Comparison between Spaniards and Colombians by current situation (student, employee, or welfare recipient) in the different subscales of the instruments used.

**Current situation**	**Instrument**	**Dimensions**	**Spain**	**Colombia**	P***	Effect size*
Student	Well-being scale	Self-acceptance	25.77 ± 6.02	25.40 ± 5.45	0.178	–0.049
		Positive relationships	27.88 ± 6.11	23.96 ± 5.74	0.000**	–0.321
		Autonomy	34.61 ± 6.96	33.97 ± 6.28	0.165	–0.050
		Control over the environment	24.97 ± 5.02	25.86 ± 4.84	0.040	0.075
		Personal growth	32.99 ± 4.99	32.73 ± 5.30	0.679	–0.015
		Purpose in life	26.36 ± 5.74	27.74 ± 5.53	0.001**	0.120
		Total score	172.61 ± 25.65	169.68 ± 25.14	0.091	–0.062
	EDATVA	Self-organization	18.84 ± 3.54	20.23 ± 3.07	0.000**	0.193
		Context analysis	20.59 ± 3.62	20.02 ± 3.41	0.007	–0.098
		Critical thinking	19.11 ± 3.73	20.30 ± 3.46	0.000**	0.160
		Sociopolitical involvement	13.66 ± 4.59	13.57 ± 4.72	0.722	–0.013
		Total score	72.20 ± 10.84	74.14 ± 10.56	0.033	0.078
Employee	Well-being scale	Self-acceptance	25.51 ± 6.27	25.41 ± 4.89	0.374	–0.057
		Positive relationships	28.78 ± 5.38	24.13 ± 5.18	0.000**	–0.416
		Autonomy	36.17 ± 6.81	34.58 ± 6.38	0.028	–0.141
		Control over the environment	25.89 ± 5.13	26.54 ± 4.81	0.545	0.039
		Personal growth	34.13 ± 4.63	32.68 ± 5.39	0.012	–0.160
		Purpose in life	27.41 ± 5.49	28.29 ± 5.02	0.261	0.072
		Total score	177.90 ± 24.56	171.64 ± 22.73	0.027	0.285
	EDATVA	Self-organization	19.70 ± 3.41	21.07 ± 3.26	0.001**	0.208
		Context analysis	21.21 ± 2.77	20.52 ± 3.69	0.189	–0.084
		Critical thinking	17.85 ± 3.74	20.06 ± 3.82	0.000**	0.278
		Sociopolitical involvement	12.10 ± 3.99	13.61 ± 4.67	0.039	0.132
		Total score	70.87 ± 8.68	75.27 ± 10.78	0.002**	–0.386
Welfare recipient	Well-being scale	Self-acceptance	22.66 ± 5.92	22.98 ± 4.64	0.942	0.006
		Positive relationships	25.03 ± 6.21	22.60 ± 5.38	0.012	0.433
		Autonomy	32.76 ± 8.36	31.56 ± 6.60	0.273	0.191
		Control over the environment	22.72 ± 5.89	24.09 ± 4.65	0.241	–0.204
		Personal growth	31.30 ± 5.94	30.29 ± 5.17	0.309	0.178
		Purpose in life	25.05 ± 6.53	25.96 ± 4.89	0.442	0.066
		Total score	159.53 ± 28.80	157.49 ± 19.15	0.410	0.144
	EDATVA	Self-organization	19.15 ± 4.63	20.18 ± 3.56	0.393	0.072
		Context analysis	20.11 ± 4.57	18.98 ± 3.72	0.046	–0.168
		Critical thinking	16.33 ± 5.76	17.60 ± 4.34	0.305	0.086
		Sociopolitical involvement	14.66 ± 5.52	14.38 ± 4.35	0.647	–0.039
		Total score	70.26 ± 15.59	71.14 ± 11.58	0.772	0.024

*Rosenthal’s R for all cases except those in which the *t*-test was applied to independent groups. Cohen’s *d* was used in these cases. **Significant considering Bonferroni’s adjustment. ***Mann–Whitney *U* test for all cases except for well-being and EDATVA total scores among employed participants, as well as for the subscales of positive relationships, autonomy, environmental mastery, personal growth, and total well-being score among welfare recipients. In these cases, the *t*-test was applied for independent groups.

## Discussion and Conclusion

The present study sought to examine the differences in psychological well-being between groups of Colombian and Spanish young people based on the dimensions proposed by Ryff. Autonomy was also taken into account, however, unlike in Ryff’s definition, we considered autonomy as a construct involving cognitive and decision-making processes in relation to other people besides oneself. The study showed the existence of significant differences between the Spanish sample and the Colombian sample on the Ryff Psychological Well-being Scale, with higher scores in the sample of Spanish youth, which reveals that the interpretation of this construct could be mediated by the country of residence and sociocultural factors (Lacomba and Cloquell, 2017; Uribe et al., 2018; Alivernini et al., 2019).

An intrinsic analysis of the scores obtained using the different elements of Ryff’s Scale showed higher scores for the Colombian group in the dimensions of purpose in life and domain over the environment, where they achieved an average score above the Spanish group. On the other hand, the scores obtained by the Spanish sample were higher in the dimensions of personal growth, autonomy, and positive relationships, and the self-acceptance dimension was found to be equivalent between both groups.

In this regard, following Crous (2017), it could be argued that the perception of well-being in each dimension would be, for each subject, a consequence of their individual trajectories, not something derived exclusively from their country of residence but from the idiosyncrasies of the social provision present in the national environment as well as the influence of values existing in each sphere. However, cultural factors have an enormous impact on an individual’s sense of autonomy, as well as the prevalent values and guiding principles in an individual’s cultural context, which shape their global perception of right and wrong and the different roles that they are expected to take on throughout their lives, one of which is the process through which young people separate from their parents (Parra et al., 2015; Blanco et al., 2019; Cabas et al., 2019). Psychological well-being is also harmonized by the influence of the environment and, especially, by the support received during the process through which young people access their autonomy (Greeson et al., 2015; Kouros et al., 2017) and the socialization process (Lun and Bond, 2016; Alonso-Stuyck et al., 2018).

Concerning the EDATVA, the mean score for the Spanish group was 71.80 (SD = 10.95), and 74.02 for the Colombian group (SD = 10.47). For Prioste et al. (2019), the social circle in which an individual exists represents an outline for the process through which he or she moves toward the achievement of full autonomy. According to Glynn and Mayock (2019) and Isakov and Hrnčić (2018), the strength of a young person’s family environment translates into differences in their psychological well-being; hence the need to develop interventions specifically designed to provide stability during people’s transition to adulthood. Similarly, authors such as Fousiani et al. (2014); Liga et al. (2017), Schofield et al. (2017), and Dutra-Thomé et al. (2019) consider that the possibility of achieving autonomy and independence is shaped by certain contextual variables that affect the configuration of the expectations forged by each individual throughout their life; this requires assessing whether the lack of equality during this transition has any effect on the way in which people decide on their long-term goals. Further analysis of the dimensions measured by the EDATVA showed that the Colombian sample presented higher scores in the critical capacity and self-organization dimensions, and the mean scores of the context analysis and sociopolitical involvement dimensions were very similar for both samples.

Comparisons between Colombian and Spanish young people based on the different subscales of the well-being and autonomy scales show the presence of a statistically significant difference in the dimension of positive relationships, in which the mean was higher for young Spaniards, whereas Colombians scored higher in purpose in life. Statistically significant differences were also observed in self-organization and critical thinking, as well as in the overall score EDATVA score; these three scores were higher for the Colombian sample. The acquisition of autonomy and the development of positive relationships with others play a central role in the psychological health and well-being of young people, but age modulates the intensity of this relationship (Inguglia et al., 2015; Lun and Bond, 2016). Autonomy can be analyzed from an individual perspective, which is understood as the individual’s capacity to make decisions that differ from their parents’ and from a collective perspective (Gao and McLellan, 2018). Similarly, according to Volkova et al. (2018), perceived support and the possibility of maintaining positive relationships with the environment are essential, both while the person is being raised and afterward.

On the other hand, there is a widespread consensus in the literature on the inequality of opportunities for people to achieve autonomy; these difference is related to one’s social group or gender, among other factors (Inguglia et al., 2015; Van der Kaap-Deeder et al., 2017; Dickens, 2018; Pinkerton and McCrea, 2018). These factors will influence with greater or lesser intensity depending on the environment studied, which could explain the differences observed in the level of autonomy between Spanish and Colombian youth, in addition to interacting with individual barriers that reduce the subject’s ability to access autonomy within of the same analysis scenario (Brim et al., 2019). In addition, it should be borne in mind that the knowledge about the problems individuals face in achieving their autonomy is limited, despite the indirect relevance of these on psychological well-being (Dutra-Thomé et al., 2019).

Along the same lines, Van der Kaap-Deeder et al. (2017) have stated that psychological well-being is facilitated in an environment where the individual can develop autonomy and exercise the ability to self-regulate emotions and behavior. For these authors, context has a large effect on psychosocial adjustment, and individuals who are given the opportunity to experience freedom and autonomy usually show psychological well-being (Van der Kaap-Deeder et al., 2015; Isakov and Hrnčić, 2018), which is associated with quality of life (Krabbenborg et al., 2017).

The possible differences between boys and girls from Spain and Colombia were analyzed. Sex-based differences were significant in the different dimensions; specifically, the Spanish average was found to be higher in the dimension of positive relationships among boys, whereas the rest of the dimensions of the well-being scale self-acceptance, autonomy, environmental mastery, personal growth, and purpose in life were very similar for both Spanish and Colombian boys. As suggested by Cabas et al. (2019), the differences may be due to the different role played by support networks in each context, which according to the authors, are more necessary for Colombian population because their opportunities require the exploitation of personal contacts in order to obtain help and meet individual expectations, which is clearly lower for young Spaniards, for whom autonomy toward subjective goals is a distinctive feature (Meléndez et al., 2018).

Concerning the EDATVA, Colombians achieved higher scores in the dimensions of self-organization and critical thinking. EDATVA results for Colombian girls were similar to those obtained by boys; they obtained higher mean scores than Spanish girls in self-organization and critical thinking, whereas Spanish girls scored better in the context analysis dimension.

In their study, Maurya and Ojha (2017) identified similar scores among young people from the same country, demonstrating the influence of context on the configuration of the trend experienced by both boys and girls; the slight differences in certain dimensions that can be explained by the different gender approaches, as interpreted by Salleh and Mustaffa (2016) or Xi et al. (2018). Other studies have shown that gender affects the level of psychological well-being: it has been shown that women enjoy less psychological well-being than men after adolescence (Akhter, 2015; Glynn et al., 2016; Sun et al., 2016; Twenge and Martin, 2020). These differences, quantifiable by psychometrics, could be derived from cognitive style and coping style.

In our study, the mean values obtained by the different subscales of the two instruments used (well-being scale and EDATVA) varied as a function of the current situations of the study participants. In this regard, the differences found between young people who were studying and those who were not in each country were statistically significant. The largest difference was observed in the dimension of positive relationships, which was higher for the Spanish sample. It should be highlighted that, except in the context analysis and socio-political involvement dimension, the mean among Spanish young people was lower in all the dimensions examined by the EDATVA. Regarding young people who were working and those who were not, the data showed that Spaniards scored higher in the dimension of positive relationships, as in the case of students and non-students. Similarly, Colombian young people obtained higher scores in the all EDATVA dimensions, except for the context analysis dimension. No statistically significant differences were observed between Spanish and Colombian welfare recipients.

In line with these results, Schofield et al. (2017) considers that the achievement of autonomy depends on the environment in which the young person develops, and as shown by the results of the present study, a person’s country of residence can represent a shortfall of resources that may decrease their ability to become adults. Cahill et al. (2016) and Glynn and Mayock (2019) have also stated that the construction of quality relationships should be an active ingredient in the design of effective interventions; therefore, it should be key in addressing the demands of young people who are on the road to independent life. This process may require a review of the social and communicative skills of the health-care staff who carry out the interventions to provide a solid background for developing the abilities and resources needed by people in this transition.

Studies such as those by Mota and Matos (2015); Vinayak and Judge (2018), and Schofield et al. (2017) stress that young people’s resilience is affected by their relationships with their social environment, their upbringing, and their current support; therefore, resilience differs as a function of one’s social support model. This mechanism has an impact on how identity is constructed and on how psychological and emotional needs are perceived throughout life.

Thus, these results provide clues to the role of contextual factors on the development of constructs such as psychological well-being and autonomy in young people who are in transition to adulthood. Consistent with studies such as Rodríguez’s (2015) on constructs associated with subjective well-being, the existence of a common core to constructs such as psychological well-being and autonomy can be proposed, as well as the effect of contextual aspects typical of an individual person’s social context, its institutions, and available life choices. The evaluation of contextual factors specific to the studied national populations were out of the scope of the present study. Future research on these topics should be geared toward a broader assessment of these aspects. Certain studies have already taken in this direction, such as the compilation by Gaxiola and Palomar (2016), which presents a regional overview of the construct of psychological well-being focused on specific aspects in countries such as Mexico, Colombia, Puerto Rico, and Cuba; an article by Domínguez (2008) which analyzes the trajectories of adult life among young Spaniards in comparison with other European populations; or the study by Bontempi (2003), which examines autonomy trajectories among young people in specific cases in Spain, Italy, and France. Another limitation of the present study is that only three conditions were considered for young people: study, work, and protection; clearly, many young people present different conditions than those covered by these three categories, and they are outside the scope of the present analysis. It is also important to evaluate the effects of COVID-19 on levels of psychological well-being and autonomy among young people, and to identify possible contextual changes in the expectations of young people and their capacity to adapt to change. Additionally, this study object could be associated with resilience, which could have a moderating effect on scale scores.

## Data Availability Statement

The raw data supporting the conclusions of this article will be made available by the authors, without undue reservation.

## Ethics Statement

The studies involving human participants were reviewed and approved by Ethics Committee of the Santo Tomás University (Bogotá, Colombia) and the Ethics Committee of the National University of Distance Education (Madrid, Spain). Written informed consent to participate in this study was provided by the participants’ legal guardian/next of kin.

## Author Contributions

All authors listed have made a substantial, direct and intellectual contribution to the present study, and approved it for publication.

## Conflict of Interest

The authors declare that the research was conducted in the absence of any commercial or financial relationships that could be construed as a potential conflict of interest.
